# Supplementary material for: Sparse transformer with local and seasonal adaptation for multivariate time series forecasting
Source: Sci Rep. 2024 Jul 10;14:15909. doi: 10.1038/s41598-024-66886-1 (PMC11237001; doi:10.1038/s41598-024-66886-1)
Supplement: Supplementary file 1 — Supplementary Information. [file 41598_2024_66886_MOESM1_ESM.pdf]

Sparse Transformer with Local and Seasonal Adaptation for Multivariate Time Series Forecasting

Supplementary Material

Yifan Zhang[1,*], Rui Wu[2], Sergiu M. Dascalu[1], and Frederick C. Harris, Jr.[1]

[1] Department of Computer Science and Engineering, University of Nevada, Reno, NV 89557, USA.

[2] Department of Computer Science, East Carolina University, NC 27858, USA.

[*] yfzhang@nevada.unr.edu

Contents

1 Datasets Details **3**

2 Detailed Computation of the Dozerformer **3**

3 More Experimental Results **5**

3.1 The quantitative results in MASE 5

3.2 Visualization of forecasting results 5

References **8**

1 Datasets Details

Table S1 shows the characteristics of benchmark datasets. The details of nine benchmark datasets utilized for the evaluation of methods are summarized as follows:

- ETT[1]: The ETT (Electricity Transformer Temperature) serves as a vital indicator for long-term electric power infrastructure deployment. Informer[1] gathered data on electricity transformers, encompassing seven key indicators such as oil temperature and useful load, from two counties in China. Among these, ETTh1 and ETTh2 datasets consist of 17,420 samples, recorded at hourly intervals. On the other hand, ETTm1 and ETTm2 datasets encompass 69,680 samples, with measurements taken every 15 minutes.

- Traffic[2]: This dataset sourced from the California Department of Transportation, comprises road occupancy rate measurements, ranging from 0 to 1, gathered from 862 freeway sites within the San Francisco Bay area. This publicly available data spans over a decade. Lai[2] meticulously collected hourly data over 48 months (2015-2016), resulting in a total of 17,544 data samples.

- Electricity[3]: This dataset, available from the UCI Machine Learning Repository, comprises the electricity consumption of 321 clients, measured in kWh at hourly intervals from 2012 to 2014, resulting in a total of 26,304 data samples.

- Weather[4]: This dataset encompasses 21 meteorological indicators, with recordings captured at 10-minute intervals over a year in Germany, resulting in a total of 52,696 samples.

- Exchange-Rate[5]: This dataset spans a period of 27 years, from 1990 to 2016, and includes daily exchange rates for eight major world economies: Australia, Britain, Canada, Switzerland, China, Japan, New Zealand, and Singapore. In total, there are 7,588 data samples available.

- ILI[6]: This dataset comprises patient data with seven indicators spanning from 2002 to 2021, with weekly sampling, resulting in a total of 966 samples. Notably, this dataset stands out due to its unique forecasting horizon setting.

The datasets were divided into distinct subsets: training, validation, and test sets. Specifically, ETTh1, ETTh2, ETTm1, and ETTm2 were divided in ratios of 0.6, 0.2, and 0.2, respectively. The other five datasets were allocated with a division ratio of 0.7, 0.1, and 0.2.

2 Detailed Computation of the Dozerformer

Given MTS input $X \in \mathbb{R}^{I \times D}$, the first step is to decompose it into seasonal and trend components[3–5] as follows:

$$
\begin{aligned}
\mathbf{X}_t &= \text{mean}\left(\sum_{i=1}^{n} \text{AvgPool}\left(\text{Padding}\left(\mathbf{X}\right)\right)_i\right) \\
\mathbf{X}_s &= \mathbf{X} - \mathbf{X}_t
\end{aligned} \tag{1}
$$

[1] https://github.com/zhouhaoyi/ETDataset
[2] http://pems.dot.ca.gov
[3] https://archive.ics.uci.edu/ml/datasets/ElectricityLoadDiagrams20112014
[4] https://www.bgc-jena.mpg.de/wetter/
[5] https://github.com/laiguokun/multivariate-time-series-data
[6] https://gis.cdc.gov/grasp/fluview/fluportaldashboard.html

Table S1. Statistics of the benchmarking datasets

Dataset	Series	Unit	Size	Length
ETTh1	7	1 hour	17,420	2 years
ETTh2	7	1 hour	17,420	2 years
ETTm1	7	15 minutes	69,680	2 years
ETTm2	7	15 minutes	69,680	2 years
Traffic	862	1 hour	17,544	24 months
Electricity	321	1 hour	26,304	36 months
Weather	21	10 minutes	52,696	1 year
Exchange-Rate	8	1 day	7,588	27 years
ILI	7	1 week	966	20 years

Where $\mathbf{X}_s \in \mathbb{R}^{I\times D}$ and $\mathbf{X}_t \in \mathbb{R}^{I\times D}$ are seasonal and trend-cyclical components, respectively.

A simple linear layer is utilized to model the trend component and generate trend component predictions.

$$\mathbf{X}_t^{pred} = \text{Linear}\left(\mathbf{X}_t\right) \tag{2}$$

where $\mathbf{X}_t^{pred} \in \mathbb{R}^{O\times D}$ is the prediction for trend component. The Linear operation directly generates O future trend values by projecting from I historical trend values for each variable in the MTS data.

The transformer encoder-decoder pair utilizing the Dozer attention is employed as the seasonal model. The DI embedding[6] is utilized to embed the raw MTS into feature maps and partition them into patches.

$$\begin{aligned} \mathbf{X}_{emb} &= \text{Conv}\left(\mathbf{X}_s\right) \\ \mathbf{X}_{pat} &= \text{Patch}\left(\mathbf{X}_{emb}, \mathbf{X_0}, p\right) \end{aligned} \tag{3}$$

where the $\mathbf{X}_s \in \mathbb{R}^{1\times I\times D}$ is the seasonal components of input, $\mathbf{X}_{emb} \in \mathbb{R}^{c\times I\times D}$ represents c feature maps embedded by a convolutional layer with kernel size of 3×1. The Patch procedure divides the time series into $N_I = \lceil I/p \rceil$ non-overlapping patches of size p, yielding $\mathbf{X}_{pat} \in \mathbb{R}^{c\times N_I\times p\times D}$. The $\mathbf{X_0}$ is zero-padding when the input sequence length I is not divisible by the patch size p. The Dozerformer adheres to a channel-independent design, where each variable is individually inputted into the transformer. Consequently, we combined the feature map dimension and the patch size dimension to form the transformer's input $\mathbf{X}_{pat}^d \in \mathbb{R}^{N_I\times(p\times c)}$.

The transformer encoder and decoder employ the Dozer attention as follows:

$$\begin{aligned} Q,K,V &= \text{Linear}\left(\mathbf{X}_{pat}^d\right) \\ \text{Attention}\left(Q,K,V\right) &= \text{Softmax}\left(Q\overline{K}^T/\sqrt{d_k}\right)V \end{aligned} \tag{4}$$

where the $\overline{K}$ is the subset of keys selected by the characteristics of datasets. Note that the $\mathbf{X}_{pat}^d$ is one variable of the MTS data, following the channel-independent design of patchTST[7]. We follow the canonical multi-head attention utilizing the attention mechanism presented in Equation 4 as follows:

$$\begin{aligned} Q_h,K_h,V_h &= \text{Linear}\left(Q,K,V\right)_h \\ \mathbf{H}_h^d &= \text{Attention}\left(Q_h,K_h,V_h\right) \\ \mathbf{H}^d &= \text{Linear}\left(\text{Concat}\left(\mathbf{H}_1^k,\cdots,\mathbf{H}_h,\cdots\right)\right) \end{aligned} \tag{5}$$

Please note that the multi-head attention, as presented in Equations 4 and 5, differs in cross attention as its Query is derived from the decoder's input. Here, $\mathbf{H}^d \in \mathbb{R}^{N_O \times (p \times c)}$ represents the transformer output, where $N_O = \lceil O/p \rceil$ denotes the number of patches of the decoder. Subsequently, the Dozerformer separates the feature dimension and patch size dimension, resulting in $\mathbf{H}^d \in \mathbb{R}^{c \times N_O \times p}$. The output for all variables is obtained by concatenating the outputs of individual variables, denoted as $\mathbf{H} \in \mathbb{R}^{c \times N_O \times p \times D}$.

Then, a Conv layer with a kernel size of 1×1 is utilized to reduce the number of feature maps from c to 1 as follows.

$$\mathbf{X}_s^{pred} = \text{Conv}(\mathbf{H}) \tag{6}$$

where $\mathbf{X}_s^{pred} \in \mathbb{R}^{O \times D}$ is the predictions for the seasonal components. Lastly, the seasonal component and trend component are summed elementwisely as follows to generate final predictions.

$$\mathbf{X}_{pred} = \mathbf{X}_s^{pred} + \mathbf{X}_t^{pred} \tag{7}$$

where $\mathbf{X}_{pred} \in \mathbb{R}^{O \times D}$ is the predictions for the future O time steps of the D variables in the MTS data.

3 More Experimental Results

3.1 The quantitative results in MASE

Table S2 illustrates the quantitative comparison results between the proposed Dozerformer and baseline methods using the Mean Absolute Scaled Error (MASE) metric.

3.2 Visualization of forecasting results

As depicted in Figure S1, we visualized the forecasting results of Dozerformer and four baseline methods on the ETTh1 dataset at horizon 96. Generally, all methods successfully capture the trend and seasonality. However, as the forecasting horizon extends to 720, the task becomes more challenging as shown in Figure S2. Notably, Autoformer exhibits a distinct gap compared to the ground truth, while the remaining methods, recognized as recent state-of-the-art approaches, demonstrate closely aligned performance.

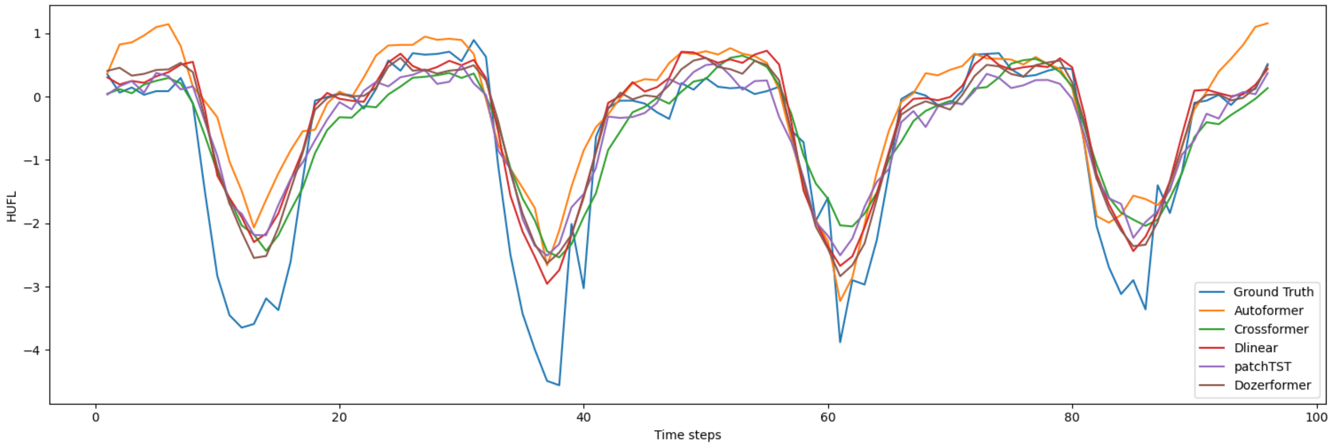

Figure S1. Visualizations of forecasting results on ETTh1 dataset at horizon 96.

Table S2. Comparison of quantitative results (MASE) on nine datasets for forecasting horizons $O \in \{96, 192, 336, 720\}$ (For ILI, $O \in \{24, 36, 48, 60\}$). **Bold** and underlined highlight the best and second-best results, respectively.

Methods		Dozerformer	PatchTST	DLinear	Crossformer	MICN	Pyraformer	FEDformer	Autoformer	Informer
Metric		MASE	MASE	MASE	MASE	MASE	MASE	MASE	MASE	MASE
$ETTh_1$	96	**0.542**	0.561	0.559	0.604	0.563	0.642	0.587	0.858	1
	192	**0.564**	0.585	0.567	0.664	0.585	0.611	0.611	0.929	1.08
	336	**0.576**	0.591	0.595	0.740	0.630	0.619	0.625	0.991	1.08
	720	**0.608**	0.619	0.648	0.888	0.661	0.693	0.670	1.034	1.14
$ETTh_2$	96	**0.780**	0.798	0.836	0.862	0.886	1.795	0.940	1.414	3.613
	168	**0.792**	0.807	0.883	0.959	0.875	1.683	0.928	1.443	4.082
	336	0.788	**0.755**	0.915	1.116	0.889	1.739	0.958	1.470	3.612
	720	0.835	**0.816**	1.065	1.384	0.905	2.169	0.916	1.514	3.143
$ETTm_1$	96	**0.500**	0.520	0.515	0.544	0.563	0.560	0.630	0.766	0.858
	192	**0.517**	0.536	0.528	0.565	0.560	0.626	0.639	0.778	0.969
	336	**0.531**	0.554	0.545	0.602	0.581	0.606	0.649	0.926	1.231
	720	**0.562**	0.576	0.577	0.652	0.617	0.737	0.672	0.993	1.128
$ETTm_2$	96	**0.758**	0.780	0.792	0.838	0.814	1.411	0.875	1.545	1.381
	192	**0.770**	0.797	0.816	1.013	0.832	1.771	0.884	1.814	1.517
	336	**0.788**	0.802	0.834	0.946	0.856	1.513	0.892	2.060	2.163
	720	**0.813**	0.827	0.905	1.053	0.866	1.618	0.892	3.120	2.877
Traffic	96	**0.223**	0.230	0.261	0.286	0.297	0.278	0.339	0.433	0.362
	192	**0.224**	0.235	0.264	0.289	0.309	0.265	0.343	0.429	0.348
	336	**0.233**	0.241	0.270	0.285	0.306	0.273	0.349	0.428	0.383
	720	**0.256**	0.260	0.287	0.296	0.319	0.285	0.348	0.431	0.430
Electricity	96	**0.232**	0.234	0.250	0.284	0.287	0.254	0.325	0.474	0.389
	192	**0.247**	0.252	0.262	0.300	0.304	0.279	0.331	0.466	0.406
	336	**0.264**	0.269	0.277	0.316	0.312	0.383	0.342	0.460	0.409
	720	0.299	**0.297**	0.308	0.329	0.328	0.433	0.364	0.456	0.450
Weather	96	**0.749**	0.779	0.933	0.901	0.866	0.912	1.165	2.188	1.511
	192	**0.801**	0.825	0.965	0.962	0.893	0.899	1.150	2.136	1.863
	336	**0.802**	0.834	0.943	0.979	0.905	1.523	1.124	2.227	1.547
	720	**0.816**	0.847	0.918	0.903	0.911	1.375	1.086	2.370	1.880
Exchange	96	1.072	1.068	**1.035**	1.198	1.193	2.169	1.418	5.637	3.836
	192	1.016	1.068	**1.013**	1.093	1.190	1.753	1.314	3.982	3.096
	336	**0.961**	1.090	1.045	1.027	1.131	1.813	1.262	2.959	2.616
	720	**0.867**	1.043	0.882	0.966	1.095	1.408	1.234	1.770	1.923
ILI	24	0.507	**0.443**	0.635	0.653	0.549	0.697	0.740	1.182	0.985
	36	**0.441**	0.461	0.511	0.566	0.488	0.653	0.573	1.078	0.778
	48	0.475	**0.453**	0.569	0.585	0.522	0.679	0.599	1.144	0.817
	60	0.529	**0.469**	0.653	0.661	0.553	0.778	0.689	1.252	0.932

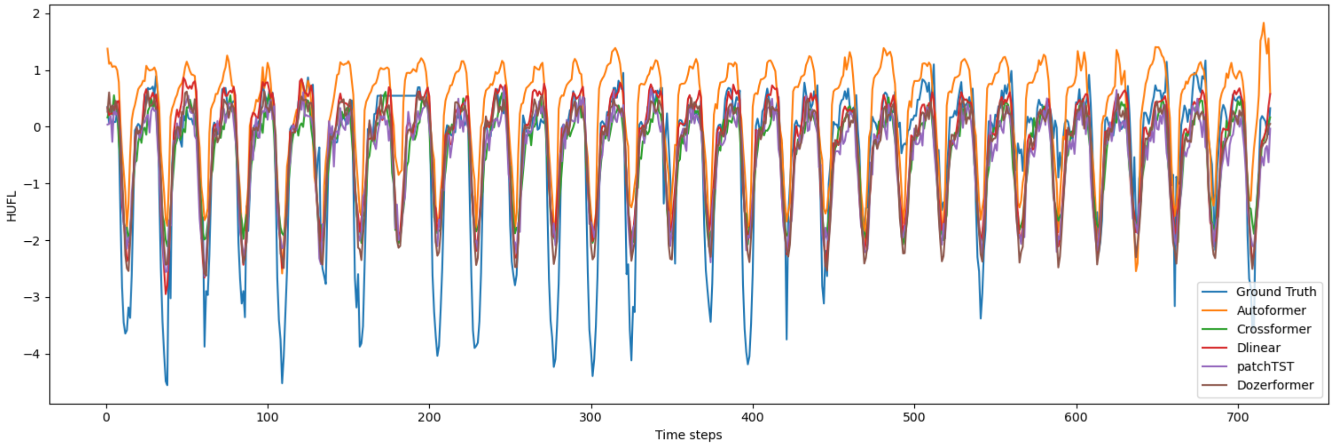

Figure S2. Visualizations of forecasting results on ETTh1 dataset at horizon 720.

References

1. Zhou, H. *et al.* Informer: Beyond efficient transformer for long sequence time-series forecasting. In *The Thirty-Fifth AAAI Conference on Artificial Intelligence, AAAI 2021, Virtual Conference*, 11106–11115 (AAAI Press, 2021).

2. Lai, G., Chang, W.-C., Yang, Y. & Liu, H. Modeling long-and short-term temporal patterns with deep neural networks. In *The 41st international ACM SIGIR conference on research & development in information retrieval*, 95–104 (2018).

3. Wu, H., Xu, J., Wang, J. & Long, M. Autoformer: Decomposition transformers with Auto-Correlation for long-term series forecasting. In *Advances in Neural Information Processing Systems* (2021).

4. Zhou, T. *et al.* FEDformer: Frequency enhanced decomposed transformer for long-term series forecasting. In *Proc. 39th International Conference on Machine Learning (ICML 2022)* (2022).

5. Zeng, A., Chen, M., Zhang, L. & Xu, Q. *Are Transformers Effective for Time Series Forecasting?*, 11121–11128 (AAAI Press, 2023).

6. Zhang, Y., Wu, R., Dascalu, S. M. & au2, F. C. H. J. Multi-scale transformer pyramid networks for multivariate time series forecasting (2023). 2308.11946.

7. Nie, Y., H. Nguyen, N., Sinthong, P. & Kalagnanam, J. A time series is worth 64 words: Long-term forecasting with transformers. In *International Conference on Learning Representations* (2023).
